# Intimate partner violence during pregnancy and quality of life in Southern Brazil: a cross-sectional study, 2022

**DOI:** 10.1590/S2237-96222024v33e2023993.en

**Published:** 2024-04-05

**Authors:** Vanessa Iribarrem Avena Miranda, Fernanda de Oliveira Meller, Antônio Augusto Schäfer, Jacks Soratto, Cristiane Damiani Tomasi, Carolina de Vargas Nunes Coll, Susana Cararo Confortin

**Affiliations:** 1 Universidade do Extremo Sul Catarinense, Programa de Pós-Graduação em Saúde Coletiva, Criciúma, SC, Brazil; 2 Universidade Federal de Pelotas, Programa de Pós-Graduação em Epidemiologia, Pelotas, RS, Brazil

**Keywords:** Pregnant Women, Violence Against Women, Primary Health Care, Intimate Partner Violence, Cross-Sectional Study, Mujeres Embarazadas, Violencia contra las Mujeres, Primeros Auxilios, Violencia de Pareja, Estudio Transversal, Gestantes, Violência contra a Mulher, Atenção Primária à Saúde, Violência por Parceiro Íntimo, Estudo Transversal

## Abstract

**Objective:**

To analyze the association between intimate partner violence during pregnancy (IPVP) and quality of life (QOL).

**Methods:**

A cross-sectional study was conducted with pregnant women receiving care in Primary Health Care in the municipality of Criciúma, Santa Catarina state, Brazil, in 2022; QOL was assessed in the physical, psychological, social relationship and environmental domains using WHOQOL-Bref instrument; IPVP was evaluated by means of the World Health Organization Violence Against Women; Crude and adjusted linear regression analyses were performed.

**Results:**

A total of 389 pregnant women were evaluated; IPVP was observed in 13.6% of cases; in the adjusted analysis, IPVP remained associated with physical, psychological and social relationship domains; pregnant women who experienced IPVP had a reduction in their QOL score by 9.77, 11.07 and 8.95 points, respectively, when compared to those who did not experience IPVP.

**Conclusion:**

IPVP was associated with poorer QOL in the physical, psychological and social relationships domains. Health services equipped to address and prevent violence against pregnant women are essential.

## Study contributions

**Main results:**

Intimate partner violence (IPV) was observed in 13.6% of pregnant women and was associated with poorer quality of life in the physical, psychological and social relationship domains.

**Implications for services:**

The results emphasize the need for an intersectoral approach in addressing the issue, with specialized healthcare centers for situations of violence integrated with social assistance and public security.

**Perspectives:**

Development of intersectoral policies and actions that strengthen existing ones and ensure social and healthcare assistance to pregnant women victims of violence and their children, given the negative impact of IPVP on quality of life.

## RESUMO

**Objetivo:**

Analisar a associação entre violência por parceiro íntimo na gestação (VPIG) e qualidade de vida (QV).

**Métodos:**

Estudo transversal, com gestantes atendidas na Atenção Primária à Saúde, em Criciúma, Santa Catarina, Brasil, em 2022; avaliou-se a QV quanto aos domínios físico, psicológico, das relações sociais e do meio ambiente (WHOQOL-Bref); a VPIG foi avaliada pela World Health Organization Violence Against Women; foram realizadas análise de regressão linear bruta e ajustada.

**Resultados:**

Foram avaliadas 389 gestantes; a VPIG esteve presente em 13,6%; na análise ajustada, a VPIG manteve-se associada aos aspectos físico, psicológico e das relações sociais; gestantes que sofreram VPIG tiveram reduzidos 9,77, 11,07 e 8,95 pontos no escore de QV, respectivamente, quando comparadas às que não sofreram VPIG.

**Conclusão:**

A VPIG esteve associada à pior QV nos domínios físico, psicológico e das relações sociais; serviços de saúde preparados para o enfrentamento e prevenção da violência contra gestantes são essenciais.

## RESUMEN

**Objetivo:**

Analizar la asociación entre violencia de pareja durante el embarazo (VPE) y calidad de vida (CV).

**Métodos:**

Estudio transversal con mujeres embarazadas en Atención Primaria de Salud, se evaluó la CV en los dominios físico, psicológico, relaciones sociales y entorno (WHOQOL-Bref); la VPE fue evaluada por la Organización Mundial de la Salud; se realizaron análisis de regresión lineal bruta y ajustada.

**Resultados:**

Se evaluaron 389 gestantes. La VPE estuvo presente en el 13,6% de las gestantes; en el análisis ajustado, la violencia de género permaneció asociada a aspectos físicos, psicológicos y a las relaciones sociales; las mujeres embarazadas que sufrieron VPE presentaron disminución de 9,77; 11,07 y 8,95 puntos de CV en comparación con quienes no sufrieron VPE.

**Conclusión:**

La VPE se asocia con una peor calidad de vida en los ámbitos físico, psicológico y de relaciones sociales; los servicios de salud preparados para combatir la violencia son esenciales para prevenir los casos de violencia durante el embarazo.

## INTRODUCTION

Violence against women, especially intimate partner violence (IPV), is a public health problem that can negatively impact physical, mental, and sexual health in both the short and long term.^1^ According to the World Health Organization (WHO), IPV refers to behavior by an intimate partner or ex-partner that causes physical, sexual, or psychological harm – such as physical aggression, sexual coercion, psychological abuse, and controlling behaviors.^1^

IPV, the most common form of violence against women,^1^ can be even more severe during pregnancy, a period of significant physical and emotional vulnerability for women. In Brazil, the prevalence of intimate partner violence during pregnancy (IPVP) ranges from 12.0% to 33.0%.^2^ According to a multi-country study conducted by the WHO between 2000 and 2003, in the majority of IPVP cases, victims reported experiencing IPV before becoming pregnant.^1^

In Brazil, about 50% of women became victims of IPV for the first time, during pregnancy; the incidence of IPVP was 11.1%, with physical violence accounting for 31.8% of cases. The vast majority of women identify the child’s father as the perpetrator of the aggression (97.5%); and 57% of them had experienced prior assaults by the same perpetrator. Furthermore, the intensity of the aggression increased during pregnancy in 26.1% of the cases. Complications related to IPVP include miscarriage (29.2%) or induced abortion (6.7%), lack of prenatal care (12.7%) and postpartum follow-up (66.7%) among women who experienced IPVP, with significantly higher percentages when compared to women who did not experience violence.^1^

IPV may be part of an ongoing pattern of abuse; serving as a determinant of health and well-being and can directly or indirectly increase the risk of premature death due to the development of health conditions.^3^ Studies have emphasized the importance of investigating the quality of life of women subjected to IPV. However, there are gaps in addressing the specific quality of life aspects for women experiencing IPVP in Brazil.^4,5^

Quality of life is synonymous with health. It is defined by the WHO as an individual’s perception of various aspects of life – spiritual, physical, mental, psychological and social – and their impact on overall self-satisfaction and well-being within their cultural context.^1^

The objective of this work was to analyze the association between IPVP and women’s quality of life.

## METHODS

This was a cross-sectional, population-based study conducted with pregnant women receiving care at all 48 primary healthcare centers (PHC) in the municipality of Criciúma, between April and December 2022. Criciúma is located in the southernmost part of the state of Santa Catarina, with a population of approximately 217,965, representing a population density of 815.87 inhabitants/km^2^, and a local human development index (HDI) of 0.788.^6^

The target population of this study was comprised of pregnant women aged 18 years and older, receiving care in the third trimester of pregnancy and undergoing prenatal care at one of the 48 PHCs in the municipality. Pregnant women facing difficulties understanding the questionnaire, of a different nationality or considered high-risk were excluded from the study. Additionally, pregnant women who reported not having any type of intimate relationship during the current pregnancy were also excluded.

The sample size calculation was performed using the OpenEpi software, taking into consideration the average annual number of pregnant women undergoing prenatal care in PHC (n = 1,517) and the following criteria: 95% confidence level, 80% statistical power, and an unknown prevalence of the outcome of 50%. An additional 10% was added for losses/refusals and 15% for controlling confounding factors, resulting in a total of 384 pregnant women to be studied.

Data were collected using smartphones and the Research Electronic Data Capture (RedCap) software to access the questionnaire, which was administered in person and in a private place. The process was carried out by community health workers (CHWs) and students of the Multiprofessional Residency program at the Universidade do Extremo Sul Catarinense (Unesc), who underwent prior training using standardized methods. After this stage, the pregnant woman was invited to respond to the confidential and self-administered questionnaire covering questions related to violence, substance and drug use. Subsequently, the completed questionnaire was sealed in an envelope, ensuring the confidentiality of the information.

In order to identify eligible pregnant women (third trimester), the municipal health system known as CELK Saúde was used. This system enabled the generation of monthly reports with the gestational age of each pregnant woman for the next three months, corresponding to the third trimester of pregnancy. All pregnant women in their third trimester from April to December 2022, were invited to take part in the study. Interviews were primarily conducted at the PHCs, according to the date of prenatal visits. When necessary, interviews were also conducted at the pregnant woman’s home.

For the assessment of the “quality of life” outcome, the World Health Organization Quality of Life Assessment (WHOQOL-Bref) questionnaire was used.^7^ This instrument, previously validated for use in the Brazilian population, including pregnant women,^8^ is focused on how respondents perceive themselves at the present moment. It aims to measure the consequences of disease and health interventions on people’s quality of life. The WHOQOL-Bref comprises 26 questions, with 24 facets related to quality of life and categorized into four domains: (i) physical (e.g. activities of daily living, substance dependence and treatment, energy and fatigue, mobility, pain and discomfort, sleep and rest and work ability), (ii) psychological (e.g. acceptance of one’s own image and appearance, positive/negative feelings, self-esteem, spirituality/beliefs, learning, memory, and concentration), (iii) social relationships (e.g., personal relationships, social support, and sexual activity), and (iv) environment (e.g., physical safety, physical environment, financial resources, freedom, health care, availability and quality of the home environment, opportunities to acquire new information and skills, participation, and leisure opportunities and transportation). Quality of life assessment scores range from 0 to 100 in each domain, with higher averages indicating a better perception of quality of life. There is no cut-off point indicating ideal values.^7^

The primary exposure variable (violence against women) was assessed using the World Health Organization Violence Against Women (WHO-VAW) instrument, available for use in Brazil,^9^ consisting of ten questions: four related to psychological violence, three about physical violence, and three about sexual violence perpetrated by intimate partners. Intimate partners were defined as spouses, companions, partners, or boyfriends. The primary exposure variable was constructed by considering “yes” for those women who experienced at least one of the three types of violence assessed during pregnancy.

The socioeconomic variables used were:

a) maternal age group (in years: 18 to 19; 20 to 29; 30 and older);
b) maternal race/skin color (White, Black, mixed-race);
c) maternal schooling (in years of study: 0 to 4; 5 to 8; 9 to 11; 12 or more);
d) monthly income (in BRL: < 500.00; 500.00-1,000.00; 1,001.00-2,000.00; 2,001.00-4,000.00; > 4,000.00);
e) marital status (single, married; stable union; separated); and
f) lives with a partner (yes; no).

In addition, parity (1, 2, 3, 4 or more), pregnancy planning (yes, no) and the number of prenatal visits (< 6, ≥ 6) were assessed.

**Figure 1:**
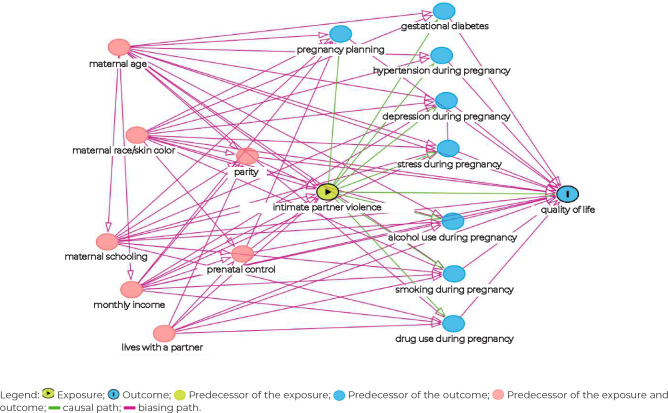
Directed acyclic graph of the association between intimate partner violence during pregnancy and quality of life

All analyses were performed using the Stata software, version 17.0. Descriptive analysis considered the absolute and relative frequencies of all categorical variables studied. For quality of life, mean comparisons were performed using the Student’s t-test (when the distribution was normal); or the Mann-Whitney/Wilcoxon Rank-Sum Test (indicated when the requirement for applying Student’s t-test was not met). Linear regression was used to analyze the exposure (IPVP) with the outcomes (quality of life domains: physical health, psychological, social relationships and environment), estimating crude and adjusted linear regression coefficients and their respective 95% confidence intervals (95%CI). The significance level used was 5%. In order to verify the minimum adjustment sets and minimize potential confounding or selection biases in the analysis, the DAGitty® software, version 3.0, was used to construct the Directed Acyclic Graph (DAG). The theoretical basis was based on bibliographic studies in the scientific literature to evaluate the relationship between violence and quality of life with covariates (Figure 1).^1,2,4,5,10-12^ The minimum adjustment sets provided by the DAGitty program included the following variables: age, race/skin color, marital status, schooling, monthly income, parity, and pregnancy planning.^13^

The study project was approved by the Research Ethics Committee of the Universidade do Extremo Sul Catarinense, Opinion No. 5,053,755, in October 2021. All pregnant women who agreed to take part in the study gave their verbal consent, using the RedCap® software.

## RESULTS

Of the pregnant women who took part in the study (n = 428), 389 (90.9%) had an intimate partner and responded to the IPV instrument (response rate of 86%). The majority were aged 20 to 29 years (53.0%), of white race/skin color (67.9%), were married or in a stable union (76.5%), had 9 to 11 years of schooling (51.9%) and, in a smaller proportion, 46.2% earned BRL 1,001.00 to 2,000.00 per month. Among all these women, 48.8% were in their first pregnancy; and the majority of them, 82.1%, had six or more prenatal visits and 59.5% had not planned the pregnancy. Regarding the types of violence experienced by these women, 12.2% were psychological, 3.1% physical and 3.1% sexual violence; 13.6% of the women who were interviewed had experienced IPVP. The mean quality of life score was 60.20 (± 16.26) for physical aspects, 66.45 (± 13.46) for psychological domain, 68.64 (± 16.10) for social relationships, and 62.03 (± 16.71) for the environment (Table 1).

**Table 1:** Sampling distribution according to the characteristics of pregnant women receiving care in Primary Health Care (n = 353), Criciúma, state of Santa Catarina, Brazil, 2022

Variables	n (%)
Age (in years)	
18-19	32 (9.1)
20-29	187 (53.0)
≥ 30	134 (37.9)
**Race/skin color**	
White	237 (67.9)
Black	39 (11.2)
Mixed-race	73 (20.9)
**Marital status**	
Single	76 (21.5)
Married/stable union	270 (76.5)
Separated/divorced	7 (2.0)
**Schooling (in years of study)**	
0-4	4 (1.1)
5-8	92 (26.0)
9-11	183 (51.9)
≥ 12	74 (30.0)
**Monthly income (in Brazilian reais - BRL)**	
< 500,00	69 (20.2)
500,00-1000,00	54 (15.8)
1.001,00-2.000,00	158 (46.2)
2.001,00-4.000,00	49 (14.3)
> 4.000,00	12 (3.5)
**Parity**	
1	105 (48.8)
2	64 (29.8)
3	30 (14.0)
4 or more	16 (7.4)
**Number of prenatal visits**	
< 6	63 (17.9)
≥ 6	290 (82.1)
**Pregnancy planning**	
No	210 (59.5)
Yes	143 (40.5)
**Psychological violence**	
No	310 (87.8)
Yes	43 (12.2)
**Physical violence**	
No	342 (96.9)
Yes	11 (3.1)
**Sexual violence**	
No	342 (96.9)
Yes	11 (3.1)
**IPVP** ^a^	
No	305 (86.4)
Yes	48 (13.6)
**Qualite of life score**	**Mean (standard deviation)**
Physical domain	60.20 (16.26)
Psychological domain	66.45 (13.46)
Social relationships	68.64 (16.10)
Enviroment	62.03 (16.71)

a) IPVP: Intimate partner violence during pregnancy.

The data in Table 2 show the mean quality of life score according to the “IPVP” outcome and the differences in each domain. The most significant difference was observed in the psychological domain: those experiencing IPVP scored 13.6 points lower. Table 3 shows the crude and adjusted analyses regarding IPVP associated with quality of life domains. In the crude analysis, IPVP was associated with quality of life in all domains (physical, psychological, social relationships and environment). In the adjusted analysis, IPVP remained associated with the physical, psychological, and social relationship domains. Women who experienced violence had, respectively, -9.77 (95%CI -14.90;4.65), -11.07 (95%CI -14.97;-7.17) and -8.95 (95%CI -13.88;-4.02) points lower in the quality of life score for physical, psychological and social relationships when compared to those who did not experience violence.

**Table 2:** Mean scores of the quality of life domains in pregnant women according to intimate partner violence during pregnancy, Criciúma, state of Santa Catarina, Brazil, 2022

IPVP^a^	Domains (scores)			
	**Physical**	**Psychological**	**Social relationships**	**Enviroment**
No	61.7	68.3	70.1	63.1
Yes	50.9	54.7	59.6	55.2
Diference	-10.8	-13.6	-10.5	-7.9
p-value	< 0.001^b^	< 0.001^c^	< 0.001^b^	0.002^b^

a) IPVP: Intimate partner violence.

**Table 3:** Crude and adjusted analysis of intimate partner violence associated with quality of life domains in pregnant women receiving care in Primary Health Care, Criciúma, state of Santa Catarina, Brazil, 2022

Variables	Crude analysis		Adjusted analysis^c^	
	**β (95%CI^b^)**	**p-value**	**β (95%CI^b^)**	**p-value**
**Physical domain**				
IPVP^a^		< 0.001		< 0.001
No	1.00		1.00	
Yes	-10.80 (-15.64;-5.96)		-9.77 (-14.90;-4.65)	
**Psychological domain**				
IPVP^a^		< 0.001		< 0.001
No	1.00		1.00	
Yes	-13.61 (-17.47;-9.75)		-11.07 (-14.97;-7.17)	
**Social relationships**				
IPVP^a^		0.001		0.005
No	1.00		1.00	
Yes	-10.46 (-15.25;-5.68)		-8.95 (-13.88;-4.02)	
**Enviroment**				
IPVP^a^		0.006		0.097
No	1.00		1.00	
Yes	-7.94 (-13.0;-2.90)		-4.17 (-9.10;0.76)	

a) IPVP: Intimate partner violence during pregnancy; b) 95%CI: 95% confidence interval; c) Adjusted for age, race/skin color, monthly income, schooling, marital status, parity and pregnancy planning.

## DISCUSSION

The prevalence of IPVP was reported in 13.6% of pregnant women. Those who experienced IPVP had lower quality of life scores for physical, psychological, and social relationship domains.

Studies on the association between violence and quality of life in women are scarce in the literature. The only study found indicated that the higher the assessment of quality of life satisfaction among women, the lower the likelihood of domestic violence occurrence, with the assessment of quality of life serving as a protective factor.^11^

Corroborating the findings of this study, a systematic review showed that poorer quality of life in pregnant women was attributed to physical factors, such as complications during pregnancy, obesity before conception, symptoms like nausea, vomiting and sleep difficulties. In addition, although this study did not assess all trimesters of pregnancy, Lagadec et al.^12^ observed a significant decrease in the quality of life related to the physical domain over the trimesters due to reduced physical activity and functional limitations. The authors also observed that the quality of life related to the psychological domain was higher during pregnancy.^12^

Similarly, a study conducted in Norway, between October 2018 and December 2019, involving pregnant women in the third trimester of pregnancy, showed that the highest quality of life score was observed in the psychological and environmental domains.^4^ However, a study carried out with women in the third trimester of pregnancy, receiving care in PHC in Rio Branco, the capital city of the state of Acre, from March to May 2011, showed a lower average for the environmental domain (60.8 ± 9.4). The highest averages were found in the physical (75 ± 11.6), psychological (73.3 ± 10.6) and social relationship (74 ± 12.4) domains.^14^

Regarding IPVP and its association with the physical domain of quality of life, it is common knowledge that women are particularly more vulnerable to IPVP, exposing them a higher risk of developing physical and mental health problems, which can even lead to disability.^15^ Similarly to the results found in this study, a systematic review demonstrated that sexual violence and domestic violence were associated with poorer quality of life in pregnant women.^12^ Research conducted in Iran between 2012 and 2013, also involving women in the third trimester of pregnancy, revealed that victims of domestic violence presented lower mean scores for physical domain of quality of life.^16^

A study conducted with women in rural Pakistan^17^ highlighted the negative impact of IPV on their quality of life and physical and psychological health. The extent of IPV effects on women’s mental health includes a higher risk of depression, anxiety, post-traumatic stress disorder, and suicide. When pregnant women are victims of violence, the consequences are amplified, increasing the risk of negative outcomes such as preterm birth, low birth weight, and small for gestational age.^18^

A systematic review, aiming to determine the worldwide prevalence of IPVP, found that 45.9% of the studies reported physical, psychological and sexual violence, with psychological violence present in 18.7% of pregnant women, rising to 23.4% in South America.^19^ IPVP is a problem that requires an intersectoral approach: it affects not only the pregnant woman, but also the fetus and the newborn, in addition to having repercussions on the pregnant woman’s social relationships.^18^

A study conducted in the state of Espírito Santo in 2017, involving 330 puerperal women, found that psychological violence perpetrated by an intimate partner was higher among younger, lower income and less educated pregnant women, who initiated sexual activity before 14 years of age. The study also indicated that pregnant women who wished to terminate their pregnancy experienced more violence during gestation.^5^

The association of IPVP with young women was also evidenced in a study conducted in the 2019-2020 biennium, with 233 pregnant women aged 10 to 49 years, in the municipality of Caxias, state of Maranhão. The same study also observed that illicit drug use by the partner remained as a factor associated with the occurrence of violence^20^ and risky sexual behaviors.^5^ Psychological violence in puerperal women is associated with partners who consume alcohol, refuse to use condoms and are not the child’s biological fathers. Physical violence, on the other hand, was associated with women whose partner were unemployed and did not agree to use condoms, in addition to an approximately nine-fold higher risk for the occurrence of sexual violence when the partner refused to use a condom.^5^

Violence during pregnancy can exacerbate difficulties in accessing healthcare adequately and regularly for pregnant women due to the control exerted by the perpetrator of intimate partner violence. This can have a negative impact on maternal and fetal health,^21^ such as preterm birth, low birth weight, and small for gestational age.^18^

As for the limitations of the study, the cross-sectional design makes it impossible to determine causality: IPVP and quality of life were measured at the same time, and it was not possible to estimate the temporality between these variables. Furthermore, it is believed that the prevalence of IPVP may be underestimated, because women are likely to hide the occurrence of violence due to the stigmatization of this condition. Finally, the types of violence (physical, psychological, and sexual) were not analyzed separately, due to the sample size of each category, which prevents data analysis with statistical power.

Among the positive aspects of the research, it is worth highlighting the representativeness of pregnant women using PHC in Criciúma, the use of validated questionnaires to measure IPV and quality of life, as well as the fact that this is the first study to estimate the association between IPVP and quality of life in Brazil. In addition, the use of the DAG enables the identification of potential confounding factors and mediating variables, reducing the likelihood of confounding or selection bias.

It can be concluded that the data showed that IPVP is associated with poorer quality of life. Taking into consideration that violence is a preventable event, intersectoral collaboration among health, education, social assistance services and public security sectors is essential in order to advance discussion on public policies and work processes aimed at preventing violence and promoting health. Given that during prenatal care, health professionals have increased contact with pregnant women, it is important to monitor their health and plan interventions in order to improve their quality of life and well-being, especially for victims of violence during pregnancy.
